# Community-led Responses for Elimination (CoRE): a study protocol for a community randomized controlled trial assessing the effectiveness of community-level, reactive focal drug administration for reducing *Plasmodium falciparum* infection prevalence and incidence in Southern Province, Zambia

**DOI:** 10.1186/s13063-017-2249-0

**Published:** 2017-11-02

**Authors:** Daniel J. Bridges, John M. Miller, Victor Chalwe, Hawela Moonga, Busiku Hamainza, Rick Steketee, Kafula Silumbe, Jenala Nyangu, David A. Larsen

**Affiliations:** 1 PATH-Malaria Control and Elimination Partnership in Africa (MACEPA), National Malaria Control Centre, Chainama Hospital College Grounds, Lusaka, Zambia; 2 Zambia Ministry of Health, Provincial Medical Office, Mansa, Luapula Province Zambia; 3 National Malaria Control Centre, Zambia Ministry of Health, Chainama Hospital, Lusaka, Zambia; 4 PATH-Malaria Control and Elimination Partnership in Africa (MACEPA), 2201 Westlake Avenue, Seattle, WA USA; 5 Syracuse University Department of Public Health, Food Studies and Nutrition, Syracuse, NY USA

## Abstract

**Background:**

Zambia is pushing for, and has made great strides towards, the elimination of malaria transmission in Southern Province. Reactive focal test and treat (RFTAT) using rapid diagnostic tests and artemether-lumefantrine (AL) has been key in making this progress. Reactive focal drug administration (RFDA) using dihydroartemisinin-piperaquine (DHAP), may be superior in accelerating clearance of the parasite reservoir in humans due to the provision of enhanced chemoprophylactic protection of at-risk populations against new infections. The primary aim of this study is to quantify the relative effectiveness of RFDA with DHAP against RFTAT with AL (standard of care) for reducing *Plasmodium falciparum* prevalence and incidence.

**Methods/design:**

The study will be conducted in four districts in Southern Province, Zambia; an area of low malaria transmission and high coverage of vector control. A community randomized controlled trial of 16 health facility catchment areas will be used to evaluate the impact of sustained year-round routine RFDA for 2 years, relative to a control of year-round routine RFTAT. Reactive case detection will be triggered by a confirmed malaria case, e.g., by microscopy or rapid diagnostic test at any government health facility. Reactive responses will be performed by community health workers (CHW) within 7 days of the index case confirmation date. Responses will be performed out to a radius of 140 m from the index case household. A subset of responses will be followed longitudinally for 90 days to examine reinfection rates. Primary outcomes include a post-intervention survey of malaria seropositivity (*n* = 4800 children aged 1 month to under 5 years old) and a difference-in-differences analysis of malaria parasite incidence, as measured through routine passive case detection at health facilities enrolled in the study. The study is powered to detect approximately a 65% relative reduction in these outcomes between the intervention versus the control.

**Discussion:**

Strengths of this trial include a robust study design and an endline cross-sectional parasite survey as well as a longitudinal sample. Primary limitations include statistical power to detect only a 65% reduction in primary outcomes, and the potential for contamination to dilute the effects of the intervention.

**Trial registration:**

ClinicalTrials.gov, ID: NCT02654912. Registered on 12 November 2015.

**Electronic supplementary material:**

The online version of this article (doi:10.1186/s13063-017-2249-0) contains supplementary material, which is available to authorized users.

## Background

Malaria remains a major public health concern, with a high associated human mortality, particularly in sub-Saharan Africa [1]. Despite these sobering figures, in the last decade multi-pronged malaria control primarily consisting of indoor residual spraying (IRS) and the use of insecticide-treated nets (ITNs) has dramatically reduced mortality and morbidity [2]. Emboldened by this progress, the newly launched Zambia National Malaria Elimination Strategic Plan (2017–2021) calls for the ambitious elimination of malaria throughout the country by 2021 using a prescribed set of vector control and treatment interventions based on epidemiologic thresholds [3]. As part of this package of interventions, enhanced malaria surveillance including reactive case detection, has been expanded to cover a large portion of Southern Province [4], an area with a documented decline in malaria prevalence to below 10% in children under 5 years of age during Malaria Indicator Surveys (MIS) conducted in 2006, 2008, 2010, 2012 and 2015 [5–9]. The enhanced surveillance data suggests that some areas within Southern Province are candidates for eliminating malaria and documenting zero transmission; however, questions remain about the strategies required to move from very low to zero transmission as well as how to document zero transmission.

Implementing and maintaining a high-quality surveillance and passive case detection (PCD) system throughout the target population is critical to eliminating transmission of infectious disease [10]. Even with universal access to malaria diagnosis and treatment, however, PCD will not identify asymptomatic infected individuals [11]. Reactive case detection is a method to identify and clear these hidden reservoirs, which tend to cluster spatially within a population, and around known symptomatic, malaria-infected individuals [12]. In areas of low transmission, incident malaria cases presenting to a PCD system can, therefore, be used as an indicator of local malaria transmission [13], and can be used to target a specific geographical areas where the probability of additional infected individuals is higher than in the general population [12].

In Southern Province, a reactive focal test and treat (RFTAT) strategy to reduce foci of infection has been implemented by the National Malaria Control Program since 2012 [4]. Briefly, this strategy consists of testing all individuals within 140 m of a diagnostically confirmed incident malaria case and then treating all positive individuals with artemether-lumefantrine (AL), or referring for further clinical care as appropriate. In Zambia, this strategy has been termed Step D as it comprises the community-level surveillance and response system as part of a stepwise approach to elimination [3]. Despite Step D being associated with a 30% increase in surveillance system sensitivity and a concomitant 6.5% reduction in outpatient attendance (Larsen et al. under review), questions remain about the implications of using an imperfect diagnostic test to direct treatment, the limited chemoprophylaxis provided when using AL and the overall effectiveness of the activities in reducing malaria transmission to zero.

In low-transmission settings, as many as 80% of malaria infections may not be detectable by microscopy [14] or a rapid diagnostic test (RDT) which have a similar limit of detection [15]. The current RFTAT approach in the Step D intervention misses these sub-patent infections during reactions; however, identifying and treating these sub-patent infections is likely to be key in accelerating the interruption of transmission [16]. An RFTAT-based approach will not only fail to identify and treat some malaria infections in the population living near an incident malaria case, but this approach also misses the opportunity to protect the local at-risk population around the incident malaria case through chemoprophylaxis. With malaria circulating in and around a location, chemoprophylaxis is likely an important piece for accelerating elimination. Indeed the chemoprophylaxis provided by mass drug administration (MDA) campaigns have been modeled as a principle driver for impact [17]. MDA maximizes chemoprophylactic protection while avoiding missing sub-patent malaria infections by delivering a therapeutic antimalarial dose to the entire target population [18]; and has had notable success in eliminating malaria from island populations, e.g., Aneityum, Vanuatu [19], been widely implemented in China [20], and has shown initial success in southern Zambia [21]. Concerns over efficacy, cost and the emergence of drug resistance have tempered its use. Nevertheless, MDA has led to success with other neglected tropical diseases and a recent Cochrane review supports the exploration of MDA as another tool in the arsenal in the quest for malaria elimination [22]. Because prophylaxis is the principle driver for impact of MDA, the choice of drug in an MDA approach is crucial. Artemisinins have short half-lives that are measured in hours; therefore, partner drugs are typically selected for their longer-lasting effects [23]. Of the two drugs approved as first-line treatments in Zambia the lumefantrine component of AL has a half-life of 4–6 days [24], while the piperaquine component of DHAP has a half-life of 28 days or more [25].

This trial will investigate whether RFDA with DHAP represents a superior intervention to RFTAT with AL in clearing and maintaining a reduced parasite reservoir in human infections and ultimately in achieving and maintaining zero malaria transmission. The primary aims are to assess these interventions using confirmed malaria incidence, longitudinal follow-ups during reactive responses and endline serology profiling.

## Methods/design

### Study site

**Fig. 1:**
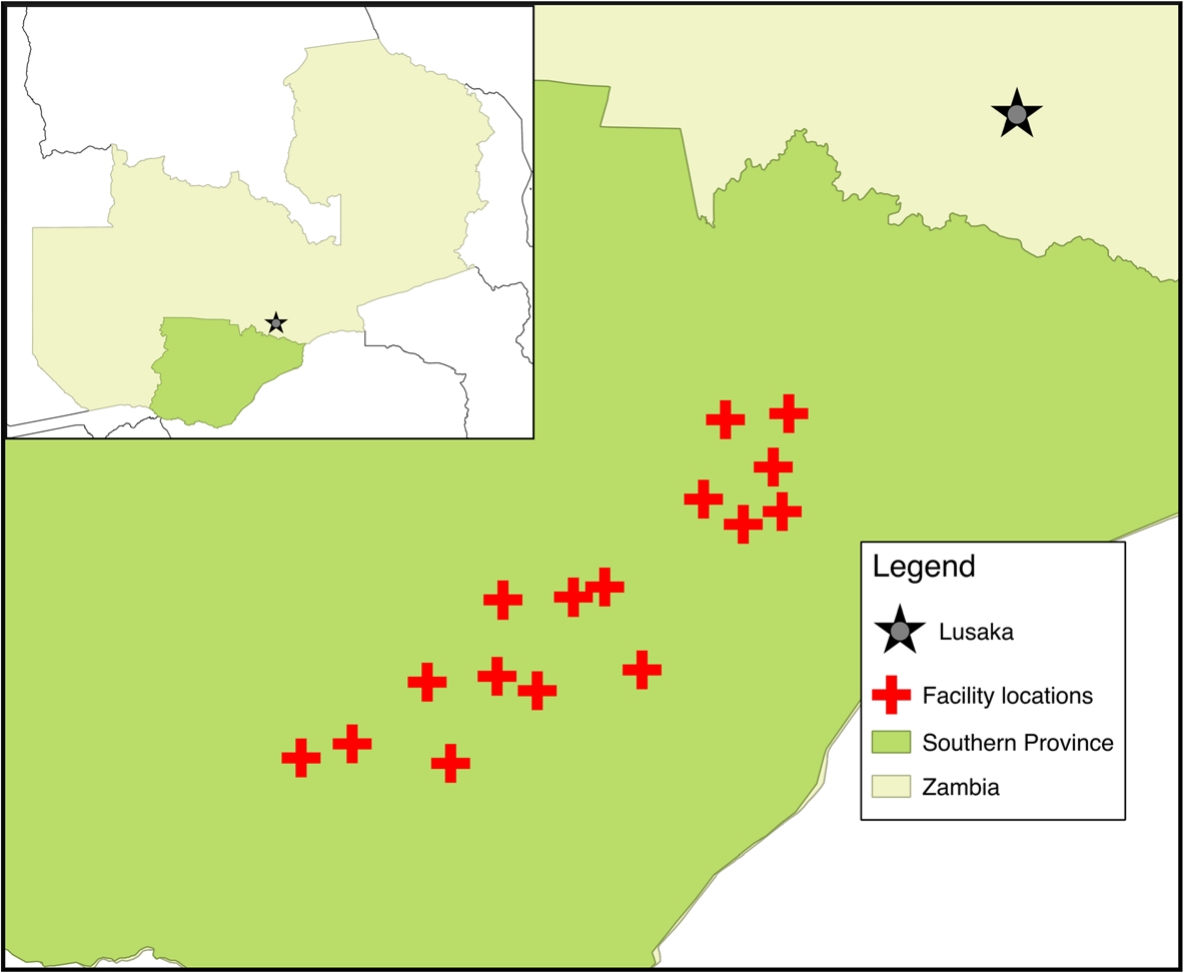
Map of the health facility catchment areas enrolled in the Community-led Responses for Elimination (CoRE) study in Southern Province, Zambia

The study will be conducted in Southern Province, Zambia in 16 health facility catchment areas (HFCA) in four districts, namely Kalomo, Monze, Mazabuka and Pemba (Fig. 1). The population living in these four districts is estimated to be approximately 130,000 living in approximately 33,000 households from 2010 census data [26]. The majority of these districts are rural and are primarily inhabited by the Tonga ethnic group, who typically live a subsistence farming lifestyle. All health facilities and health posts were mapped and geographically defined prior to this study in the national health management information system.

Malaria transmission in this area is low, with an annual incidence across all 16 HFCAs of 12.7 per thousand for 2015. As with the rest of Zambia, malaria transmission is seasonal, peaking in April/May shortly after the end of the rainy season. ITN coverage, defined as at least one ITN per household, for the province is high at 77.8%. ITNs are supplemented with IRS, with much lower, but increasing levels of coverage using the organophosphate pirimiphos methyl which remains a highly effective insecticide in the area. Malaria testing and treatment is free to the public at all government-sponsored health facilities and in these rural districts access to malaria testing and treatment in the private market is minimal.

### Interventions

#### Dihydroartemisinin plus piperaquine (DHAP)

In the intervention arm, all consenting research participants will receive a treatment dose of DHAP. Sigma Tau (Rome, Italy) is currently the only Stringent Regulatory Authority licensed and currently the only World Health Organization (WHO)-prequalified manufacturer of DHAP, from whom the drug will be sourced under the brand name Eurartesim. In Zambia, DHAP is an approved alternate first-line treatment for uncomplicated malaria [27]. Consenting research participants will be offered 4 (2–10) mg/kg/day dihydroartemisinin and 18 (16–26) mg/kg/day piperaquine over the course of 3 days according to the manufacturer’s recommendations, as well as Zambian national guidelines.

#### Intervention arm: reactive focal drug administration (RFDA) with DHAP

Upon presentation of a confirmed incident malaria case, at a government health facility or health post, a volunteer community health worker (CHW) will return to the household of the incident malaria case and give treatment of DHAP to all consenting individuals living within 140 m of the incident case household who meet the drug inclusion criteria.

#### Control arm: reactive focal test and treat (RFTAT) with RDT and AL

Upon presentation of a confirmed incident malaria case, at a government health facility or health post, a volunteer CHW will return to the household of the incident malaria case and test all individuals living within 140 m for a malaria infection using a RDT. All those testing positive will be given a treatment dose of AL.

#### Directly observed treatment

Due to the reliance on volunteer CHWs for delivering treatments at the household level and because of the 3-day treatment regimen, it was determined that implementing a full directly observed treatment (DOT) regimen would not be feasible. In lieu of this, a modified DOT schedule will be employed, consisting of the initial dose, on day 1, being given under DOT, followed by a second visit on day 3. During the day 3 visit, compliance with the day-2 and day-3 doses will be recorded, treatment blister packs observed and additional instruction/encouragement given to achieve maximal compliance where needed.

#### Inclusion and exclusion criteria

**Fig. 2:**
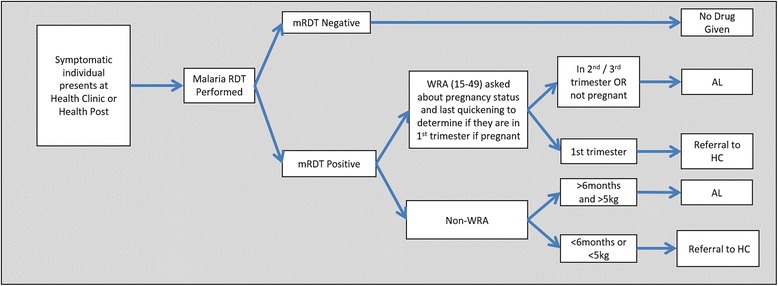
Participant flow chart enrolled during passive case detection and implemented in all arms. *WRA* (15–49 years) women of reproductive age 15 to 49 years old, *mRDT* malaria rapid diagnostic test, *HC* health center, *AL* artemether-lumefantrine

**Fig. 3:**
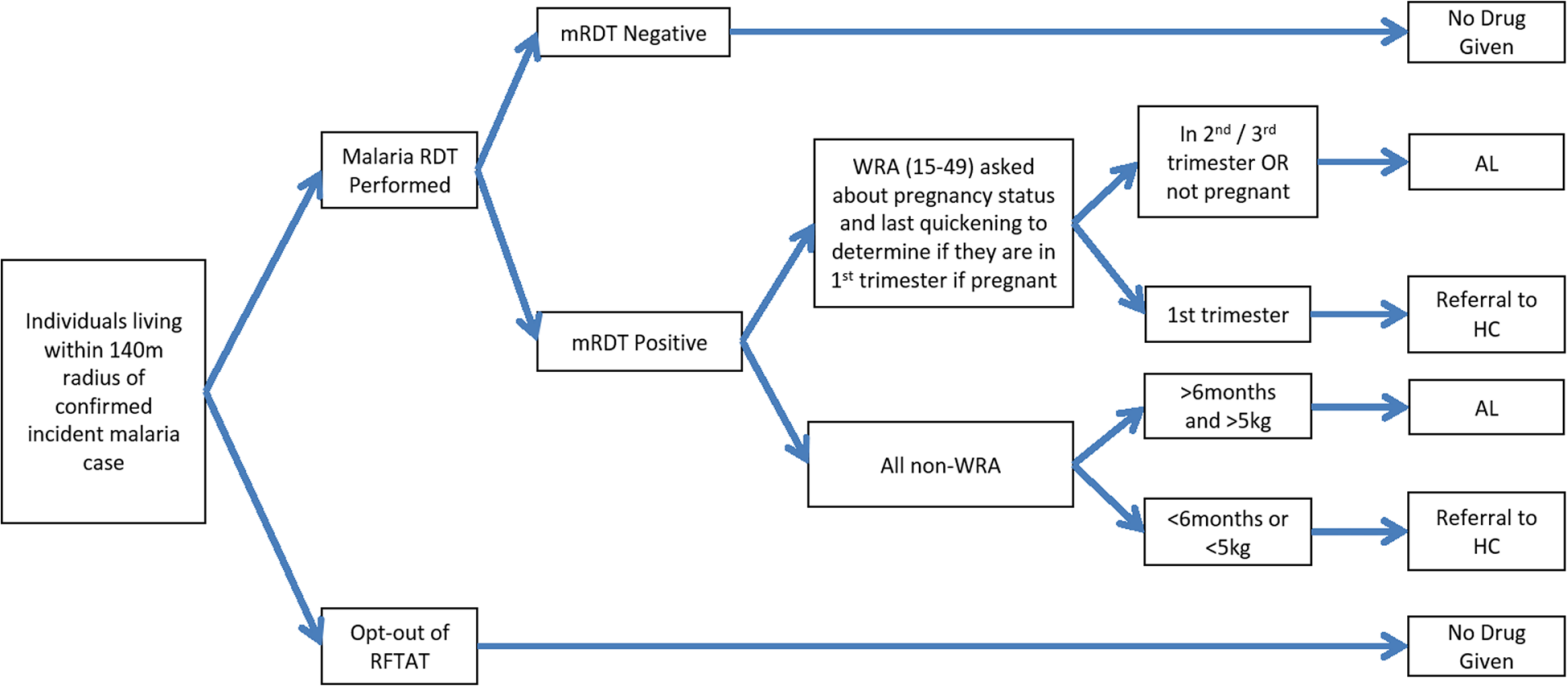
Participant flow chart to determine antimalarial treatment regimen under reactive focal test and treat (RFTAT) with AL. *WRA* (15–49 years) women of reproductive age 15 to 49 years old, *mRDT* malaria rapid diagnostic test, *HC* health center, AL artemether-lumefantrine

**Fig. 4:**
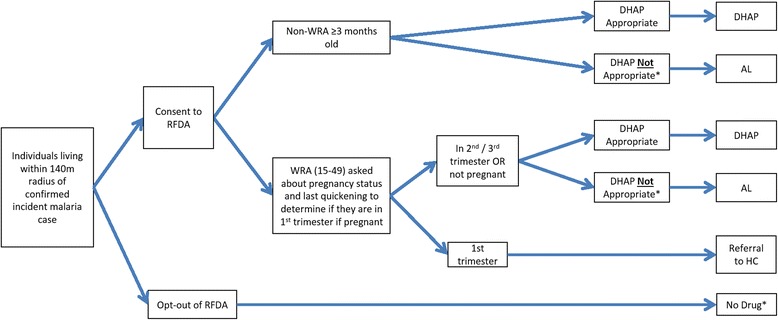
Participant flow chart to determine antimalarial treatment regimen under reactive focal mass drug administration (RFDA) with DHAP. *WRA* (15–49 years) women of reproductive age 15 to 49 years old; *mRDT* malaria rapid diagnostic test, HC health center, *AL* artemether-lumefantrine, *DHAP* dihydroartemethesin-piperaquine. *Individual will be offered standard of care (see Fig. 3)

Across both arms, case investigations will be initiated from standard PCD of treatment-seeking symptomatic individuals (Fig. 2). Based on the identification of a confirmed index case, individuals will be offered the standard of care (Fig. 3) in the control arm or offered RFDA in the intervention arm (Fig. 4). In accordance with the manufacturer’s instructions, children younger than 3 months old and pregnant women in their first trimester will be excluded from receiving DHAP. Pregnancy status during the first trimester will be determined among women aged 12 to 49 years through direct questioning, and by offering a urine-based pregnancy test for women unsure of their pregnancy status or reporting more than 5 weeks since their last menstrual period. In the intervention arm young children (aged below 3 months) and pregnant women will be offered the standard of care which consists of testing with a RDT and then an appropriate antimalarial, according to the national treatment guidelines, if the RDT is positive. Finally, any individuals found during case investigations with suspected severe malaria or other severe illness will not be enrolled in the study but will be referred to the nearest health center. Severe illness may include severe anemia, prostration, impaired consciousness, respiratory distress, convulsions, circulatory collapse, abnormal bleeding, jaundice and passing dark urine.

Patients presenting with an adverse event will be graded for severity and treated according to national guidelines for the drug that they have taken. All adverse events will be recorded and reported to the Data Safety Monitoring Board.

### Community mobilization

All of the HFCAs involved in this study are already implementing Step D, i.e., the control arm, through the CHW cadres, who are well acquainted with the process of conducting case investigations around incident malaria cases. This research will, therefore, only alter the details of the reactive response in the RFDA intervention arm. Despite these apparent small changes to the community expected response, and to encourage high levels of participation and adherence, several sensitization activities were conducted. Firstly, information about this research, the treatments and the process were shared through the Ministry of Health. Secondly, sensitization meetings were held with chiefs and their headmen to inform key community leaders and ensure that information filtered down through the traditional community leadership structures. During CHW trainings, all clinic-based supervisors and in-charges were also invited to attend to learn about the study. Information materials were adapted from another MDA study in Southern Province [28] and will be distributed during community sensitization meetings.

### Study design

A cluster randomized controlled trial will be used to evaluate the impact of sustained RFDA with DHAP versus RFTAT with AL at reducing malaria parasite seropositivity and malaria incidence at the HFCA population level, as well as reducing reinfection in the population treated during reactive case detection. Communities, or clusters, are defined by HFCAs and serve as the unit of randomization. HFCAs were selected based on having a mean monthly incident malaria case load of less than 25 (absolute count) and a mean test positivity during historical Step D reactive case detection of 5% or more. A total of 16 health facilities and their catchment areas are included in the study, each of which is randomly allocated to either the control or intervention arm. Blinding is not possible with this study design due to supervision needs of CHWs. However, all analysis will be blinded by the use of a concealed study code for the study arm.

Evaluating an intervention in a pre-elimination setting is exceptionally challenging. By definition, transmission and, therefore, outcomes to be evaluated are extremely low. The small numbers in potential outcomes means that traditional methodologies typically employed to evaluate malaria interventions, e.g., with human landing catches to measure the entomological inoculation rate or sampling the parasite prevalence in a human population, are not appropriate. Measuring transmission in a pre-elimination setting, therefore, requires new tools. Molecular genotyping, e.g., with a barcode [29], can expand the amount of data that can be extracted from a single identified infection. For example, relationships between individual infections with the same or a related barcode can be inferred. Furthermore, at the population level, the overall genetic diversity can inform transmission levels [30]. This rich granular data may allow local transmission patterns to be dissected, but it still requires samples to be collected during an infection. Additionally, serology looks at the cumulative exposure of an individual to malaria. The production of an immune response to any antigen is a function of antigen exposure, the ability of an antigen to provoke an immune response (antigen immunogenicity), and the decay rate of the antibody response. Assuming that all individuals in a population share the same exposure risk, and that the immunogenicity of different parasite strains is comparable, then the likelihood of converting from being seronegative to seropositive increases with age. By calculating the age-specific seroconversion rates at two time points, one can measure whether transmission has increased or decreased [31]. Furthermore, by using multiple antigens with different decay rates or immunogenicities [32, 33], windows of exposure can be defined. Ultimately, serology can be, and has been, used to document malaria elimination, e.g., in Greece [34]. Finally, serology can be used to model the entomological inoculation rate [33], which otherwise would not be possible to define given the levels of transmission in the area.

### Primary outcome measures

Primary outcomes used to measures the impact of the intervention versus the control are:

1. *Plasmodium falciparum* seropositivity in children stratified into younger (aged ≥ 1 month to < 2 years) and older (aged ≥ 2 years to < 5 years) age groups. Seropositivity is defined as the proportion of seropositive children out of all children enrolled in that age group
2. Total and confirmed outpatient (OPD) malaria case incidence and inpatient (IPD) malaria case incidence among all ages: defined as the number of OPD and IPD malaria confirmed and suspected cases per person per year, as ascertained from the routine rapid reporting system; facility catchment population size estimates will be used for the exposure denominator
3. *P. falciparum* clearance rate among the cohort of individuals enrolled in a longitudinal study, defined as the percentage of individuals with a *P. falciparum* infection (based on polymerase chain reaction (PCR)) enrolled on day 30
4. *P. falciparum* reinfection rate among a cohort of individuals enrolled in a longitudinal study, defined as the percentage of individuals with a *P. falciparum* infection (based on PCR) enrolled on day 90
5. Complexity of infection (COI) found during reactive responses to incident malaria infections
6. Complexity of infection (COI) in endline survey

### Data collection procedures for measurement of outcomes

For all data collection procedures, consent will be obtained from each participant prior to enrollment in the trial.

#### Diagnosis of *Plasmodium falciparum* infections

During case investigations CHWs will use RDTs to diagnose the presence of *P. falciparum* parasite infections for all individuals enrolled in the study. Furthermore, for a subset of research outcomes we will confirm RDT results using PCR, as described below. The RDTs used in this trial are manufactured by Standard Diagnostics Inc. (Gyeonggi-do, Republic of Korea) under the brand name SD Bioline Malaria Antigen *P.f*. These RDTs detect the Histidine-Rich Protein 2 (HRP-2) antigen of *P. falciparum* and are approved by the Zambian Ministry of Health for use in case management. For PCR analyses we will collect capillary blood from finger pricks onto filter paper. These dried blood spots will be analyzed as part of the longitudinal cohort analysis.

#### Endline survey

A cross-sectional random survey of households will be performed at the end of the high-transmission season (April/May) at the end of the 2-year study, i.e., in 2018. The aim will be to obtain a representative sample of children (aged ≥ 1 month to < 5 years) to determine the first primary outcome (seropositivity). A complete enumeration and geo-referencing of all households in the study area, with a 3-km buffer applied around the location of each health facility will serve as the sampling frame. This buffer will mitigate contamination between the control and intervention arms. Using satellite enumerated households [35–37] as a sampling frame, a simple random sample of households will then be drawn within each of the 16 HFCAs (equal allocation sampling), to obtain the total sample size of 2820 households. Selected households will be visited by trained survey data collectors who will administer a standardized survey questionnaire, adapted from the standard national malaria indicator survey questionnaire [6], loaded onto mobile devices. A dried blood spot will be prepared from a finger prick for all children sleeping in the household the previous night where consent has been given, and will be used to measure seropositivity by ELISA (Enzyme-linked Immunosorbent Assay) and parasite prevalence by PCR.

#### Routine health Information

In 2011, a routine aggregate reporting system utilizing District Health Information System 2 (DHIS2) was established. Reporting to this system is performed via data-enabled mobile phones with data available to be viewed through a password-protected website. At the health facility, data are reported weekly and include reporting on clinical and laboratory-confirmed malaria cases, total outpatients, RDT stock levels, etc. At the level of the CHW health post, data is reported monthly and includes information on RDT-confirmed cases from both passive and reactive case detection. From this database we will generate a time-series dataset to evaluate the second primary outcome. We will standardize total malaria cases and confirmed malaria cases two ways for each HFCA: by estimated mid-year populations and monthly total outpatient attendance. The Ministry of Health together with local partners have been implementing a standard data quality auditing process in these districts since 2013 to improve the overall quality of surveillance data from health facilities and health posts.

#### Longitudinal cohort

**Fig. 5:**
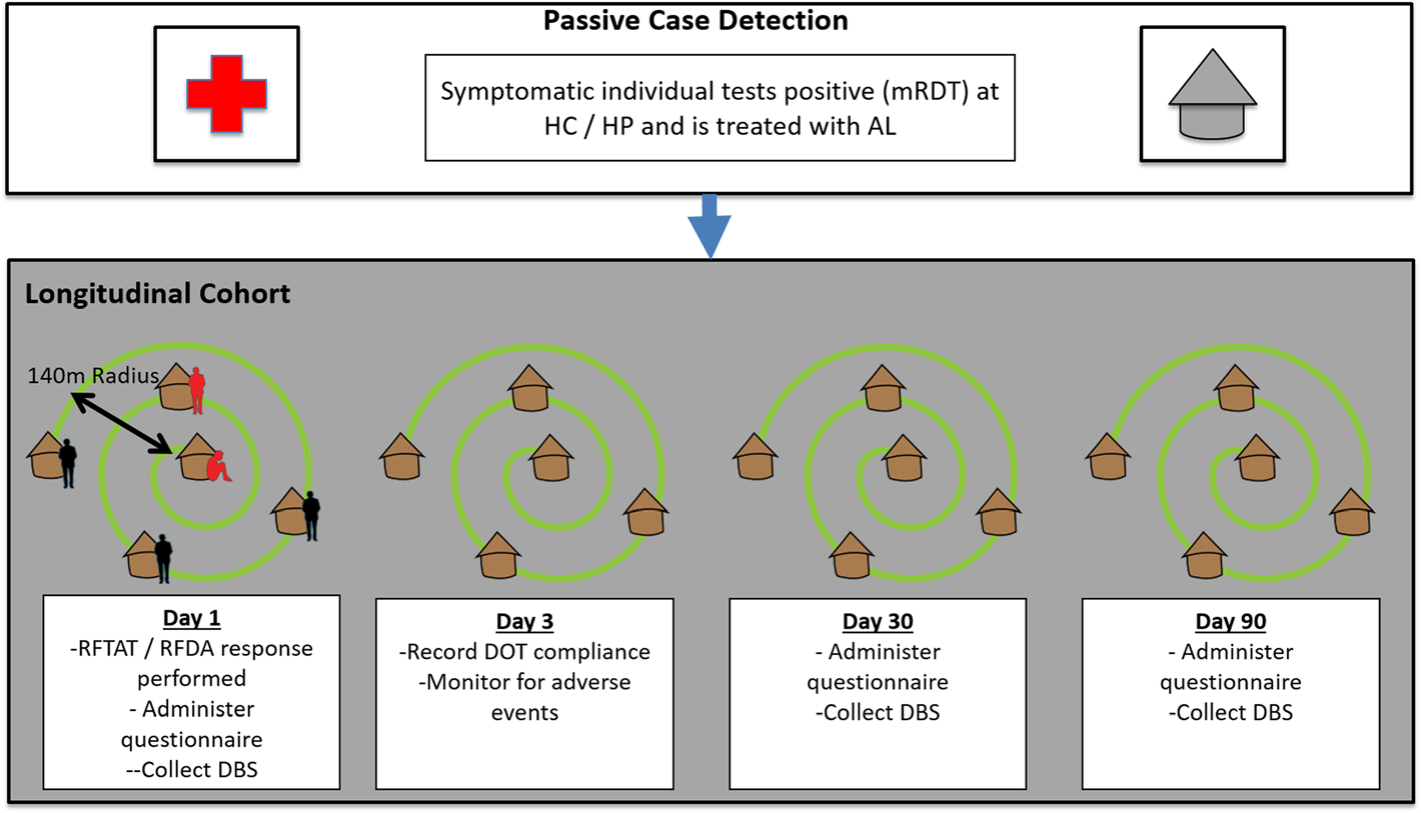
Summary schematic of interactions for participants enrolled in the longitudinal study. *mRDT* malaria rapid diagnostic test, *HC* health center, *HP* health post, *AL* artemether-lumefantrine, *DBS* dried blood spot, *DOT* direct observed therapy

A number of the reactive responses will be selected for longitudinal follow-up, using a convenient sample with, where possible, equal geographical representation. Individuals enrolled in this cohort will be visited on days 1, 3, 30 and 90 by study teams in addition to the responding CHW. With the exception of day 3, where only the modified DOT compliance will be recorded, a standard questionnaire will be administered and a dried blood spot collected at each time point from each participant (see Fig. 5 for a summary of interactions and activities at each time point). Dried blood spots will be used to detect the presence of malaria parasites by PCR to evaluate primary outcomes 3 and 4. To encourage retention, where available, PCR results from previous visits will be shared. PCR-positive, RDT-negative individuals will be offered an additional RDT test on subsequent visits.

#### Cost data

Detailed tracking of program expenditures on the establishment, production and delivery components for both the RFDA intervention and RFTAT control, including the value of time spent by the CHWs and other staff, will be used to calculate the financial cost of the intervention. This data will be collected from a range of inputs including program records, invoices, budgets, expenditure reports, etc.

### Sample size and statistical power

Using the Hayes and Bennett formula for estimating the number of clusters we found that a total of 16 clusters (eight intervention and eight control) will provide adequate power to measure the effectiveness of the intervention [38]. We estimated sample size requirements for the endline survey, and also provide an estimate of power for the longitudinal cohorts.

#### Endline survey

An inter-cluster variation of 0.5 was found in a previous study evaluating the use of mass screening and treatment campaigns in the districts adjacent to Lake Kariba [39]. To calculate sample size requirements we assumed our outcome of seropositivity would be equal to or greater than 5% (this is the assumed parasite prevalence by RDT in the same population). Including eight health centers per intervention arm would allow for detection of a 64.5% relative difference between intervention arms with 80% power and 95% specificity (decrease from 5% seropositivity to 1.78%).

#### Longitudinal cohort

Two separate outcomes are considered from longitudinal cohorts, that of parasite clearance during a reaction and parasite reinfection. These outcomes will be measured using PCR, and assuming an 8% parasite prevalence rate as measured by RDT we presume approximately 15% parasite prevalence rate as measured by PCR. No data were available to estimate a coefficient of variation for these outcomes, so we conservatively use a coefficient of variation of 0.5 for the power calculations. With these assumptions the 16 clusters will allow us to measure a 65.4% and 67.4% decrease in parasite clearance 3 days following the case investigation and reinfection 90 days following the case investigation, respectively, with 80% power and 95% specificity.

### Statistical analysis plan

To estimate the impact of the RFDA intervention for primary outcome one (malaria parasite exposure through seropositivity) we will employ a mixed-effects regression model with the health center as a random intercept. This approach will account for correlated data at the HFCA level and covariates will include environmental factors, socio-economic status, housing construction, education level, and demographic measures of the population as well ITN coverage and IRS.

Confirmed malaria incidence at health centers (primary outcome two) will be analyzed on an intention-to-treat basis regardless of the location of the incident cases’ household. We will use a Poisson mixed-effects regression model with the health center as a random intercept to evaluate the effectiveness of the intervention on malaria incidence. In case of over dispersion we will use a negative binomial distribution model. The model will include pre- and post-intervention as well as intervention or control covariates and their interaction to create a difference-in-differences analysis. We will also include environmental factors.

*P. falciparum* infection, as measured by PCR-positivity during reactions, will be analyzed using a Poisson mixed-effects regression model with the CHW as a random intercept to determine primary outcome three. In case of over dispersion in the data, we will use a negative binomial regression model. The number of people tested in each reaction will serve as the offset, and various environmental and socio-demographic factors will be included in addition to the intervention or control fixed effect.

To analyze primary outcome 4, *P. falciparum* infection as measured by PCR-positivity following reactions will be analyzed in two ways: first using a Poisson mixed-effects regression model with the CHW as a random intercept and second using a time-to-event analysis with the CHW as a shared frailty. For both analyses various environmental and socio-demographic factors will be included in addition to the intervention or control fixed effect.

### Cost analysis

Costs will be calculated using an ingredients approach that involves enumerating both the quantity of specific inputs (e.g., hours spent, number of RDTs used, etc.) and the time spent during the intervention based on recent methods used in Southern Zambia [40]. As the analysis is intended to be incremental, existing infrastructure and recurrent inputs that would be present in the absence of the intervention will not be costed. The emphasis of the cost analysis is on determining the cost of RFDA or RFTAT alone, and not assessing the cost of training CHWs in the diagnosis and treatment of malaria nor in the follow-up visits to areas receiving reactive responses. Cost-effectiveness will be measured through incident malaria cases averted as measured through the difference in malaria incidence at health centers in the control versus the intervention arm.

### Quality control and secondary studies

#### Importation of infections

All malaria infections will be geo-referenced during data collection. Spatial variation in the genomic complexity and diversity will be estimated using the Geneland [41] package in R [42] which uses Bayesian methodology to identify clusters of individuals with similar genotypes while accounting for spatial dependence of geo-referenced data. The procedure is capable of analyzing multi-variate gene outcomes including single-nucleotide polymorphisms (SNPs). The number of separate genetic populations in the study area will be estimated and mapped; migrants (*P. falciparum* infections dissimilar genetically from others in nearby space) will be classified as imported malaria cases without onward transmission and will also be identified and mapped. The influence of a number of environmental factors on the spatial distribution of malaria parasites will be estimated. The Enhanced Vegetation Index available through remotely sensed data will provide an indicator of adult mosquito habitat [43, 44]. A digital elevation model will be used provide an indicator for the propensity of an area to harbor mosquito breeding sites [45, 46]. We aim to utilize spatio-genetic methods that have been utilized in various aspects of ecology, but have not yet been applied to malaria epidemiology to understand transmission dynamics in the two arms [47].

Genetic complexity, defined as the number of genetically unique parasites found within health center and health post catchments, will be compared between RFDA and RFTAT areas using a Poisson or linear regression depending upon the number of genetically unique parasites found. A number of factors will be tested including proximity of the area to the main road, wealth of the area, vector control coverage of the area, age and demographics of the individuals living in the area, as well as environmental factors such as vegetation cover and topographical measures.

The complexity of infection (COI) will also be calculated for each infection [48] and any changes over time between the control and intervention arm identified.

### Adherence

Drug administration strategies require high coverage to achieve impact. We will measure adherence to both RFTAT and RFDA in two ways. First, we will measure a simple percent-refusal among those offered the intervention during case investigations. Second, we will compare household counts to the number of individuals available for treatment during case investigations. These factors will be an important predictor in the analyses previously described.

#### Study timeline

**Fig. 6:**
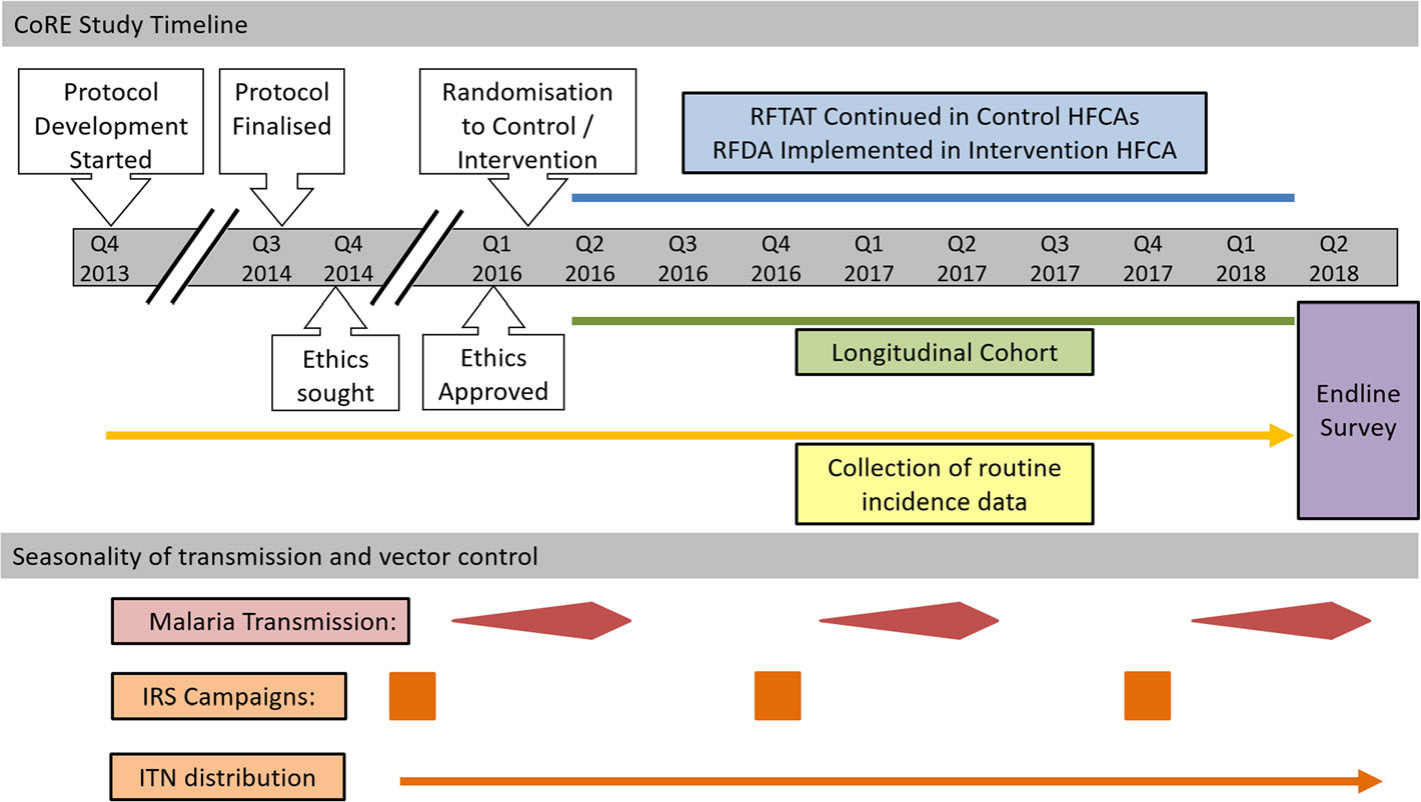
Study timeline for activities during the Community-led Responses for Elimination (CoRE) study as well as malaria transmission seasonality and vector control activities. *RFTAT* reactive focal test and treat, *RFDA* reactive focal drug administration, *HFCA* health facility catchment areas, IRS indoor residual spraying, *ITN* insecticide-treated net

A schematic depicting a timeline highlighting the key activities to be performed as part of CoRE are shown in Fig. 6. The study began enrolling participants in March 2016 after randomization of HFCAs to the intervention/control arm and training of the CHWs and health facility staff. The longitudinal study runs concurrently with the CHW-led interventions and will continue for the duration of the study. Routine data has been collected in these HFCAs since 2013 and will continue indefinitely.

The endline survey is due to be performed at the end of the peak transmission season in April/May 2018.

### Ethical Approval

Ethical approval in Zambia for this study has been obtained from the Research Ethics Committee (REC) at the University of Zambia (study reference 011-10-14) and the Zambian Medicines Regulatory Authority (trial number CT 052). Ethical approval was also obtained from the Western Institutional Review Board (IRB) (for PATH-MACEPA; study number 1155095).

## Discussion

Zambia’s ambition to eliminate malaria has been distilled into an aggressive national strategic plan that calls for the already significant progress made in malaria control to be accelerated. Effective interventions must also be scalable, sustainable and affordable in order for Zambia to achieve and maintain elimination. This study will compare the effectiveness and cost-effectiveness of RFDA to the current intervention of RFTAT with the aim of moving from very low levels of malaria transmission to hopefully zero transmission. RFDA is scalable, affordable and sustainable as demonstrated by ongoing RFTAT in Southern Province, Zambia. Data from this study will provide key evidence necessary to determine if RFDA is a tool that can help more quickly achieve the long-term goal of elimination.

This cluster randomized control trial is rigorously designed to assess both the impact of parasite clearance at the population level, through routine health facility malaria incidence data and an endline survey, as well as clearance at the level of individual reactive responses, through longitudinal cohort data. By measuring serology as well as prevalence the endline survey will not only provide evidence that transmission is increasing or decreasing, but is designed to robustly identify and document the absence of transmission. To minimize potential contamination during the endline survey households will be sampled within a 3-km buffer from the health facility.

The quality of the implementation of the intervention and control arm will be measured by target population coverage, adherence to the protocol and acceptability of the study. In-depth longitudinal cohorts will also give an idea of the reintroduction rate of *P. falciparum* malaria following RFTAT and RFDA and the environmental factors that might facilitate that. Reintroduction poses a significant threat to the endgame of malaria eradication and this study will give insight into factors affecting the rate of reintroduction at the micro scale.

A significant challenge for the study is the scarcity of outcomes (both confirmed malaria incidence and malaria parasite prevalence) in the study HFCAs. When evaluating the impact of malaria elimination strategies sample sizes become prohibitively large. With that in mind we have designed our study such that the data may be pooled with an ongoing trial in Swaziland (ClinicalTrials.gov, ID: NCT02315690). Further, we are using multiple outcomes to assess the effectiveness of the intervention (Additional file 1).

## Trial details

This trial was initially registered at ClinicalTrials.gov, ID: NCT02654912 on 12 November 2015. This manuscript is based on version 7, 2 September 2015. Funding for this study is being provided by the Bill and Melinda Gates Foundation, with PATH as the primary sponsor.

## Data management

Data collected during the endline survey and the reactive research responses will be recorded on an Android tablet. This personally identifiable data will be securely transferred over the telephone network, as soon as a data connection is available, to a central server. Access to the server will be password protected and secured according to best practice. Local copies of the database will be secured in password-protected files on password-protected computers.

Prior to analysis, data will be de-identified with the exception of geo-location codes which are necessary for specific per-protocol analyses, the absence of individual identifying information will protect subject confidentiality. Only trial investigators will have access to the complete final trial dataset.

All paper records, Consent Forms and biological specimens will be stored in a locked location.

## WHO data set

### Health condition studied

Malaria

### Intervention name

Reactive focal drug administration with dihydroartemisinin-piperaquine (DHAP).

### Intervention description

In response to the detection of a confirmed case of malaria Southern Province, Zambia, the current standard of care is to mount a reactive response, i.e., to test all individuals within a defined radius of the index cases and treat all positives with artemether-lumefantrine (AL). The intervention tested compares this standard of care, to one where all individuals living within the response radius are presumptively treated with DHAP.

### Inclusion criteria

All individuals, regardless of sex, older than 3 months.

### Study type

Interventional phase 4 study. Health facilities are the unit of randomization.

### Date of first enrollment

1 March 2016

### Target sample size

Four thousand eight hundred for endline survey

### Recruitment status

The study is ongoing, with both CHW-led and research team supported responses having started in March 2016. Enrollment is due to continue until March 2018 with the endline scheduled immediately after.

### Primary outcomes

Malaria seropositivity in children aged under 5 years after 2-year intervention within health center catchment areas

### Key secondary outcomes

1. Incidence of malaria confirmed by RDT or microscopy as measured through passive case detection at health posts and health centers over the 24-month trial
2. Parasite prevalence (by polymerase chain reaction) among individuals participating at 0, 30 and 90 days during a reactive research response
3. Cost-effectiveness of intervention in reducing the burden of malaria
4. Proportion of *P. falciparum* infections likely attributable to importation or local transmission events by parasite genotyping as well as defining the spatial distribution of different genotypes

### Data Safety Monitoring Board

A DSMB will be assembled to support the study and will be comprised of six members composed of physicians, clinical researchers, Government of Zambia medical personnel, biostatistician/epidemiologist, lay member of the public.

## Additional file

Additional file 1: SPIRIT 2013 Checklist: recommended items to address in a clinical trial protocol and related documents. (DOCX 50 kb)

## Abbreviations

AL: Artemether-lumefantrine
CHW: Community health worker
DHA: Dihydroartemisinin
DHAP: Dihydroartemisinin plus piperaquine
DOT: Directly observed therapy
HFCA: Health facility catchment area
IRB: Institutional Review Board
IRS: Indoor-residual spraying
MDA: Mass drug administration
NMEC: National Malaria Elimination Center
PCR: Polymerase chain reaction
RDT: Rapid diagnostic test
REC: Research Ethics Committee
RFDA: Reactive focal mass drug administration
RFTAT: Reactive focal test and treat
WHO: World Health Organization
WRA: Women of reproductive age
